# COVID-19 vaccine hesitancy: the five Cs to tackle behavioural and sociodemographic factors

**DOI:** 10.1177/01410768211018951

**Published:** 2021-06-02

**Authors:** Mohammad S Razai, Pippa Oakeshott, Aneez Esmail, Charles Shey Wiysonge, Kasisomayajula Viswanath, Melinda C Mills

**Affiliations:** 1 Population Health Research Institute, St George University of London, London, SW17 0RE, UK; 2 School of Primary Care Research, National Institute for Health Research, University of Manchester, Manchester, M13 9PL, UK; 3 South African Medical Research; and Faculty of Medicine and Health Sciences, Stellenbosch University, Stellenbosch,7505, South Africa; 4 Department of Social and Behavioral Sciences, Harvard T.H. Chan School of Public Health (HSPH), Harvard University, Boston, Massachusetts 02215, USA; 5 Department of Sociology, University of Oxford and Nuffield College, Oxford, OX1 1NF, UK

The global roll-out of the COVID-19 vaccine is a cause for celebration. Vaccinations are the most successful public health measure in history, saving millions of lives each year globally, preventing disease and bringing enormous societal and economic benefits.^1^ Reversing and mitigating the ongoing damage wrought by COVID-19 is largely contingent on a successful worldwide equitable vaccination strategy.^2^ An estimated 60%–70% of the world’s population needs to be vaccinated to achieve an effective herd immunity.^3,4^

One of the biggest hurdles to vaccinations is hesitancy: a delay in acceptance, or refusal despite availability. We propose five Cs to tackle vaccine hesitancy: Confidence (importance, safety and efficacy of vaccines); Complacency (perception of low risk and low disease severity); Convenience (access issues dependent on the context, time and specific vaccine being offered^5,6^); Communications (sources of information); and Context (sociodemographic characteristics) (see Figure 1).^1^

**Figure 1.**
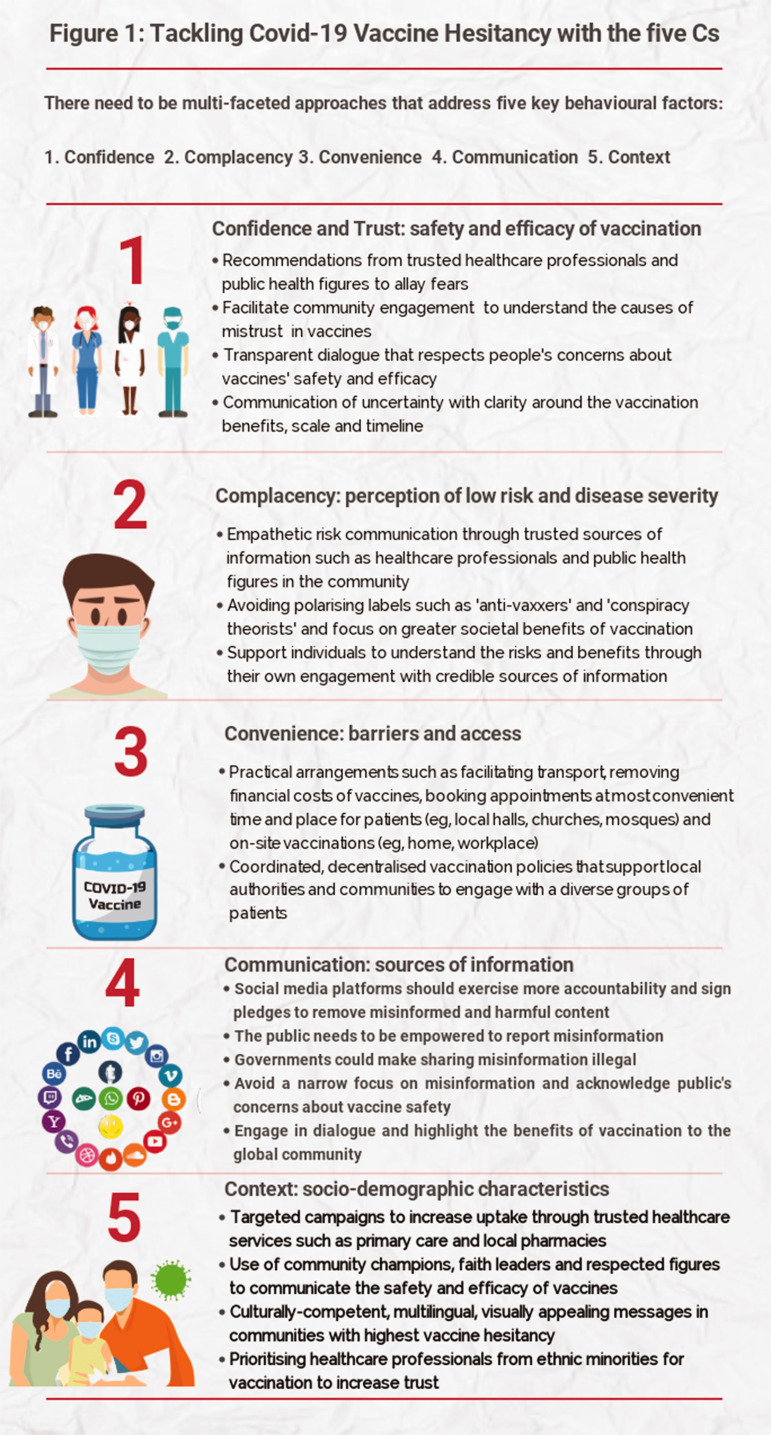
Tackling COVID-19 vaccine hesitancy with the five Cs.

## Confidence

Confidence in vaccine safety, efficacy and importance is crucial, and highlighted by recent concerns about the possible association between the AstraZeneca and Johnson & Johnson vaccines and very rare unusual blood clots such as cerebral venous sinus thrombosis.^2,7^ The public need to understand that these events are extremely rare (estimated 4/million people vaccinated), the risk of getting cerebral venous sinus thrombosis if you contract COVID-19 may be up to 10 times higher than getting it due to vaccination, and for most people the benefits of vaccine vastly outweigh the risk. Other factors affecting uptake include historic distrust along with underrepresentation of ethnic minorities in clinical trials, and religious concerns about the safety and acceptability of the vaccine. In Muslim-majority countries such as Indonesia and Malaysia, a drop in confidence in the vaccine was directly due to religious rulings of vaccines being *haram* (e.g. including unacceptable ingredients derived from pigs or containing alcohol).^1^ This year, the month of Ramadan for Muslims is between April and May. Building confidence in the vaccine also requires sensitive, non-stigmatising messages that, for example, the intramuscular injection does not nullify one’s fast (which is observed dawn to dusk). Perceptions of vaccine safety and efficacy are the strongest predictors of vaccine uptake and many vaccine-hesitant people cite concerns about safety and side effects.^8,9^ It is clearly crucial to engage in transparent dialogue that respects people’s concerns and acknowledges uncertainty.

## Complacency

Complacency is strongly associated with lower vaccine uptake. Lower perceptions of personal risk and disease severity for COVID-19 have been reported in younger people and individuals of lower socioeconomic status.^10^ As the lower age groups are being offered the vaccine, addressing complacency through repeated risk communication is crucial to facilitate informed decision making. It is important to emphasise the greater societal benefits of population level immunity and the protection it offers to those vulnerable, their families and friends.

## Convenience

Evidence points to the crucial role of well-planned and convenient vaccination delivery, emphasising the role of an easy-to-reach location and attention to financial costs of having the vaccine.^1^ High vaccination levels were reported in the US when it took place at schools^11^ and similarly a high uptake in the UK of the influenza vaccine was achieved through pharmacies and general practices.^1,12^ However, recent data from England show that more people expected a longer wait and more inconvenient vaccination than they actually experienced.^13^ Perceptions of convenience may also need to be addressed.

## Communication

According to the World Health Organization, the world is also fighting an ‘infodemic’ of ‘a few facts, mixed with fear, speculation and rumour’ which, within the context of ongoing uncertainties and knowledge gaps, has been amplified through technology and social media platforms. An excessive amount of information, rapid changes in COVID-19 information and guidance, and lack of certainty has caused misinformation to spread faster than the infection, thus creating general distrust and confusion.^14^ Misinformation feeds on people’s fears and anxieties about the pandemic to promote anti-vaccination conspiracy theories.^15^ A genuine transparent dialogue backed by community engagement is required to address the public’s concerns and build confidence. It is also important to acknowledge uncertainties. Social media platforms should exercise more accountability and remove harmful and misinformed content.^7,15,16^ Lessons learnt from previous pandemics and immunisation programmes suggest that vaccine deployment should exploit existing infrastructure such as primary care, pharmacies and trusted healthcare professionals.^12^

## Context

Context including ethnicity, religion, occupation and socioeconomic status is often overlooked. The problem starts with the term vaccine hesitancy itself. Although it has been widely used in the literature and adopted by the World Health Organization, it does not take account of the powerful structural factors such as systemic racism and access barriers which may lead to low vaccine take-up in some groups. Further, it places an emphasis on individual agency and implies a degree of blame. European data show lower intention to be vaccinated against COVID-19 among racial and ethnic minorities, those with lower education, younger people and people with previously poor compliance with recommended vaccinations,^1,17^ with corresponding poor uptake of COVID-19 vaccines in some ethnic minorities and deprived communities.^18^ This follows a historic trend^19^ in the UK, and suggests that COVID-19 has exacerbated inequalities related to ethnicity and socioeconomic status.^20^

## Conclusions

Vaccine hesitancy is complex, variable and shaped by multiple contextual factors. Most research has been conducted in high-income countries and few interventions have been found to be effective in low-income and middle-income settings.^21^ It is therefore essential that along with COVID-19 Vaccines Global Access (COVAX)^22^ – a mechanism to fairly distribute COVID-19 vaccine doses around the world – there is a concerted international effort to understand, analyse and overcome vaccine hesitancy.^23^ International organisations such as the Red Cross, Red Crescent and UNICEF have the experience and expertise to communicate risk during a crisis. Strengthening local capabilities to mobilise diverse communities by addressing the five Cs of vaccine hesitancy through tailored, appealing, culturally competent and multilingual messages is supported by evidence and could have the highest chance of success.

## Declarations

**Ethics approval:** Not applicable.

**Guarantor:** MSR

**Contributorship:** MSR conceptualised and wrote the manuscript, incorporated feedback from co-authors and finalised the manuscript. PO helped with the writing and contributed to the revision and concepts. AE, CSW, KV and MCM provided feedback, edits and contributed to the concept. The final manuscript was approved by all authors.
